# BNIP3 modulates the interface between B16-F10 melanoma cells and immune cells

**DOI:** 10.18632/oncotarget.24815

**Published:** 2018-04-03

**Authors:** Erminia Romano, Nicole Rufo, Hannelie Korf, Chantal Mathieu, Abhishek D. Garg, Patrizia Agostinis

**Affiliations:** ^1^ Laboratory for Cell Death Research and Therapy (CDRT), Department of Cellular and Molecular Medicine, KU Leuven, Leuven, Belgium; ^2^ Laboratory of Hepatology, Department of Chronic Diseases, Metabolism and Ageing (CHROMETA), KU Leuven, Leuven, Belgium; ^3^ Laboratory of Clinical and Experimental Endocrinology (CEE), Department of Chronic Diseases, Metabolism and Ageing (CHROMETA), KU Leuven, Leuven, Belgium

**Keywords:** cancer, immunogenic cell death, phagocytosis, macrophage, anticancer vaccination

## Abstract

The hypoxia responsive protein BNIP3, plays an important role in promoting cell death and/or autophagy, ultimately resulting in a cancer type-dependent, tumour-enhancer or tumour-suppressor activity. We previously reported that in melanoma cells, BNIP3 regulates cellular morphology, mitochondrial clearance, cellular viability and maintains protein expression of CD47, a pro-cancerous, immunosuppressive 'don't eat me' signal. Surface exposed CD47 is often up-regulated by cancer cells to avoid clearance by phagocytes and to suppress immunogenic cell death (ICD) elicited by anticancer therapies. However, whether melanoma-associated BNIP3 modulates CD47-associated immunological effects or ICD has not been explored properly. To this end, we evaluated the impact of the genetic ablation of BNIP3 (i.e. BNIP3^KD^) in melanoma cells, on macrophage-based phagocytosis, polarization and chemotaxis. Additionally, we tested its effects on crucial determinants of chemotherapy-induced ICD (i.e. danger signals), as well as *in vivo* anticancer vaccination effect. Interestingly, loss of BNIP3 reduced the expression of CD47 both in normoxic and hypoxic conditions while macrophage phagocytosis and chemotaxis were accentuated only when BNIP3^KD^ melanoma cells were exposed to hypoxia. Moreover, when exposed to the ICD inducer mitoxantrone, the loss of melanoma cell-associated BNIP3 did not alter apoptosis induction, but significantly prevented ATP secretion and reduced phagocytic clearance of dying cells. In line with this, prophylactic vaccination experiments showed that the loss of BNIP3 tends to increase the intrinsic resistance of B16-F10 melanoma cells to ICD-associated anticancer vaccination effect *in vivo*. Thus, normoxic vs. hypoxic and live vs. dying cell contexts influence the ultimate immunomodulatory roles of melanoma cell-associated BNIP3.

## INTRODUCTION

Skin cutaneous melanoma is a highly aggressive malignancy wherein tumour progression and metastatic dissemination are facilitated by a perpetual cross-talk between melanoma cell-autonomous oncogenic processes and melanoma-elicited immunosuppression [1, 2]. Disruption of this cross-talk is one of the reasons behind the success of targeted therapy (that inhibits oncogenic signalling) and immunotherapy (that dampens immunosuppression driven by cancer cells or the deregulated microenvironment) [3, 4]. Indeed, targeted therapy and immunotherapy have achieved remarkable success against melanoma. But, a considerable proportion of patients do exist that fail to respond to these treatments due to disparate resistance or compensatory mechanisms adopted by melanoma cells [4]. These trends have raised an impending need to unravel how the interface between melanoma cells and immune cells is regulated. Such an endeavour has important therapeutic implications since, in the future, appropriate targeting of this interface may achieve a doubly effective anti-melanoma efficacy.

Hypoxia is a crucial microenvironmental factor, which promotes immunosuppression and tumorigenesis largely through the transcriptional program coordinated by the hypoxia responsive protein, HIF1α (hypoxia inducible factor 1 alpha) [5, 6]. One of the well-established targets of HIF1α is BNIP3 (Bcl-2 [B-cell Leukaemia/Lymphoma 2]/Adenovirus E1B1] Nineteen kD Interacting Protein 3) [7]. BNIP3 is an atypical BH-3 only protein, belonging to the BCL2 family of proteins that have an important role in cell death regulation. Notably, BNIP3 has contextual pro-death and pro-autophagic roles, which likely underlie the reported cancer type-dependent effects of BNIP3 as tumour-enhancer or tumour-suppressor [8–12]. We recently found that BNIP3, which is expressed at baseline levels in melanoma cells under normoxia and is further stimulated by hypoxia, serves critical pro-melanoma functions. These include (but are not limited to) regulation of key cellular processes like migration, survival and long-term clonogenic growth [13]. Indeed, genetic ablation of BNIP3 in B16-F10 melanoma cells *in vitro* had severe consequences for actin-based cytoskeletal architecture, cellular morphology, mitochondrial clearance and viability [13]. Moreover, BNIP3 ablation reduced the overall protein levels of CD47 [13], an observation reminiscent of the effect of BNIP3 on the CD47 levels in T lymphocytes [14].

Beyond its well-known function as integrin-associated protein and intracellular regulator of G-protein signal transduction, plasma membrane-associated expression of CD47 serves as a key ‘don’t eat me’ signal. CD47 achieves this blockade of phagocytosis through interaction with the signal regulatory protein α (SIRPα) on the surface of phagocytes like macrophages [15–17]. However in cancer cells, the increased expression of CD47 can enable pro-cancerous immunosuppression by deregulating the phagocytic uptake of cancer cells by the innate immune cells [15, 16, 18, 19]. Interestingly, a recent study revealed that the expression of CD47 is transcriptionally regulated in breast cancer cells by HIF1α [20]. CD47 expression was reported to correlate with HIF1 target gene expression in breast cancer patients and ultimately associate with poor patient survival [20]. Notably, our analysis in melanoma patients also unravelled a positive correlation between *CD47* and *BNIP3* transcript levels [13], suggesting that the hypoxia-responsive protein BNIP3 may be a modulator of the phagocytic barrier in melanoma cells. CD47 is also relevant for suppression of another anti-tumorigenic immune process i.e. immunogenic cell death (ICD) [21]. ICD is a mode of cell death induced by various therapeutic approaches (including chemotherapeutics like anthracyclines), that can elicit pro-immunogenic processes through a spatiotemporally defined exodus of danger signals and cytokines/chemokines [22–24]. ICD is often counter-acted by some cancer cell-autonomous pathways that either cause deregulation of phagocytosis or disrupt the sensing of ICD by the immune cells. These include (but are not limited to), CD47 up-regulation, inability to properly surface expose (ecto-) ‘eat me’ signals like calreticulin (CALR), or reduction in chemotactic signals facilitating sensing of cancer cells dying through ICD by the immune cells [21, 25–28].

Hence in this study, we evaluated the impact of the genetic ablation of BNIP3 in melanoma cells on the most foundational immunological processes like phagocytosis and chemotactic recruitment, both *in vitro* and *in vivo*. We also evaluated whether melanoma-associated BNIP3 affects crucial determinants of chemotherapy-induced ICD such as danger signals and *in vivo* anticancer vaccination effect.

## RESULTS

### BNIP3 and hypoxia regulate the phagocytosis of B16-F10 melanoma cells by J774 macrophages

**Figure 1:**
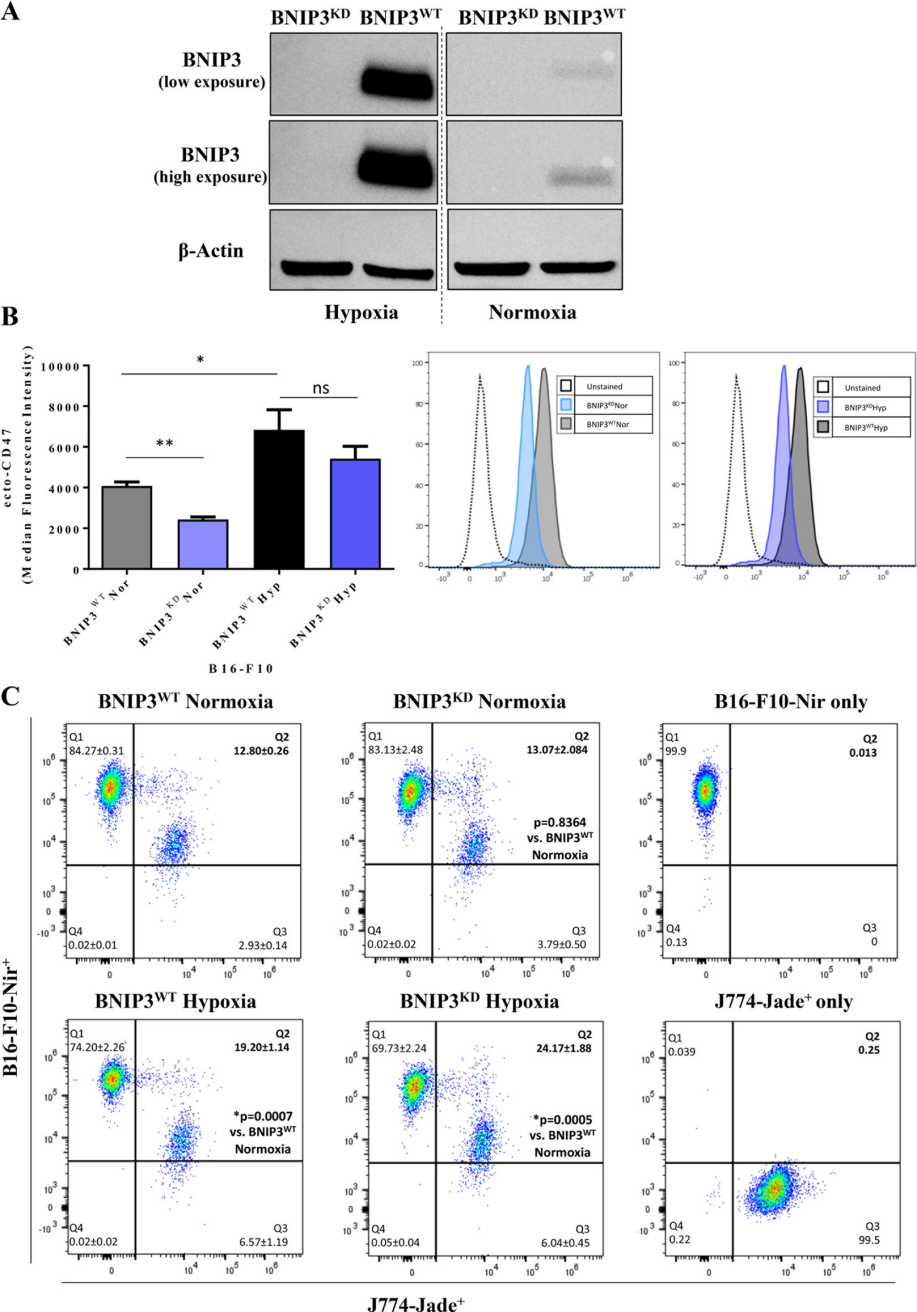
BNIP3 and hypoxia modulate the phagocytosis of B16-F10 melanoma cells by macrophages (**A**) Representative western blot for the efficiency of BNIP3 knock-down in B16-F10 cells after 24 h treatment under normoxic and hypoxic conditions (both low and high exposure are reported). Actin was used as loading control. (**B**) Flow Cytometry-based quantification (left panel) and representative histograms (right panel) of the level of surface CD47 (ecto-CD47) in B16-F10 cells (BNIP3^WT^ and BNIP3^KD^) after 24 h of culture in normoxia (Nor) or hypoxia (Hyp). Data expressed as mean ± SEM and analysed with Mann-Whitney’s *t*-test (^**^p = 0.0043, ^*^p = 0.0411, ns = not significant [*p* = 0.3052]) as indicated by the bar, *n* = 3 independent experiments). (**C**) J774-mediated phagocytosis of B16-F10 after 24 h co-incubation, based on flow cytometry data with relative gating strategy. The engulfing ability is reported in Q2 as percentage of B16-F10-Nir^+^ / J774-Jade^+^ cells. Data expressed as mean ± SD analysed with Student’s *t*-test and *p*-value, with respect to BNIP3^Nor^, is reported in Q2 quadrant (This graph is a representative of independent experiments; *n* = 3).

In order to systematically decipher the role of cancer cell-associated BNIP3 in regulating the melanoma-immune cell interface, we knocked-down the overall expression of BNIP3 via the shRNA methodology (BNIP3^KD^), in the well-established murine B16-F10 melanoma cells (Figure 1A). Additionally, to account for the prominent status of BNIP3 as a hypoxia-inducible molecule [7], we compared the effects of normoxia (20% O_2_) with hypoxia (1.5% O_2_) on the respective B16-F10 cells. Notably, the knock-down of BNIP3 was prominent under both normoxic and hypoxic conditions, which, as expected, elevated the protein levels of BNIP3 only in the B16-F10 cells expressing control shRNA (i.e. BNIP3^WT^; Figure 1A). Interestingly, BNIP3^WT^ B16-F10 cells exposed to hypoxia, significantly (^*^p = 0.0411) up-regulated the surface levels of CD47 (i.e. ecto-CD47) as compared to the ones exposed to normoxia (Figure 1B). The latter observation aligns with the published literature demonstrating the immunosuppressive effects of hypoxia [5]. Notably, BNIP3^KD^ B16-F10 cells not only exhibited a significant (^**^p = 0.0043) reduction in ecto-CD47 in normoxic conditions, as compared to BNIP3^WT^ cells (in keeping with our published report [13]); but also displayed a tendency to dampen ecto-CD47 levels under hypoxia, albeit non-significantly (Figure 1B; *p* = 0.3052).

To understand the impact of this interplay between BNIP3-hypoxia-CD47 on the phagocytic clearance of melanoma cells by macrophages, we performed *in vitro* phagocytosis experiments. These involved co-incubation of B16-F10 cell lines with the macrophage-like J774 cells followed by flow cytometric scoring of phagocytosis [29, 30]. Notably, BNIP3^KD^ B16-F10 cells, under normoxic settings, did not undergo efficient phagocytic clearance by the J774 cells (Figure 1C). This was surprising, considering the significantly reduced ecto-CD47 levels exhibited by these cells (Figure 1B). Alternatively, when the cells were exposed to hypoxia, the phagocytic clearance of BNIP3^WT^ B16-F10 cells by J774 cells was increased significantly (^***^p = 0.0007, as compared to normoxic-BNIP3^WT^ cells; Figure 1C), thereby indicating the presence of phagocytic determinants other than CD47 in this setting. Moreover, this hypoxia-stimulated phagocytosis of living melanoma cells was further potentiated by BNIP3^KD^ in a statistically significant manner (^***^p = 0.0005, as compared to normoxic-BNIP3^WT^ cells; Figure 1C). In fact, the phagocytosis of BNIP3^KD^ cells under hypoxia was also significantly higher than BNIP3^WT^ cells under hypoxia (^*^p = 0.0172, Figure 1C).

Next, we analysed the ecto-CALR levels in BNIP3^WT^ or BNIP3^KD^ melanoma cells (relative to normoxia or hypoxia). FACS analysis using an anti-CALR antibody did not reveal any significant exposure of this molecule in the above conditions (Supplementary Figure 1). This ruled out the possibility that differences in ecto-CALR might be counter-balancing the susceptibility of melanoma cells to undergo phagocytosis in the above settings. Thus, B16-F10 melanoma-specific BNIP3 ablation in combination with hypoxia, stimulates phagocytic clearance of melanoma cells by macrophages, although this does not inversely correlate with the ecto-CD47 levels.

### BNIP3 does not alter the ability of melanoma cells to recruit, polarize, or be phagocytosed by macrophages *in vivo*

In a tissue context, efficient phagocytic clearance of damaged cells at a ‘wounding site’ is carried out by local as well as newly recruited macrophages [31]. In the latter case, a ‘wounding site’ is typically flagged by various chemotactic factors, which can be sensed by macrophages for directional chemotaxis [27, 31]. Of note, B16-F10 cells have been widely reported to modulate the chemotactic recruitment of different immune cells, including macrophages [27, 32]. To this end we wondered whether BNIP3, also in light of its dominant functions in cytoskeleton regulation [13], may affect the secretion of pro- or anti-macrophage chemokines. To address this, we initially examined the release of major chemokines by BNIP3^WT^ and BNIP3^KD^ cells, relative to normoxic or hypoxic conditions, by using an antibody array followed by a volcano plot-based representation. Although to an insignificant level, BNIP3^KD^ in B16-F10 cells (either alone or in combination with hypoxia), exerted diverse (and sometimes even contradictory) effects on the secretion of various chemokines and cytokines, with disparate functionalities (Supplementary Figure 2). More specifically under normoxic conditions (Supplementary Figure 2), BNIP3^KD^ B16-F10 cells secreted increased amounts of the pro-myeloid cell chemokines i.e. CCL2 (a potent macrophage recruiting chemokine) and CXCL1 (a potent neutrophil recruiting chemokine) (Supplementary Figure 2). Yet in the same setting, some chemokines with the ability to attract myeloid cells (CX3CL1, CXCL10), macrophages (CCL3) or lymphocytes (CX3CL1, CXCL16, CXCL10) exhibited strong reduction in secretion (Supplementary Figure 2). Normoxic BNIP3^KD^ B16-F10 cells also exhibited reduction in secretion of certain pro-inflammatory cytokines like GM-CSF and IL1α (Supplementary Figure 2). Curiously, hypoxic treatment of BNIP3^KD^ B16-F10 cells largely diminished the above patterns observed with normoxic BNIP3^KD^ B16-F10 cells (except for CXCL10) (Supplementary Figure 2). However, hypoxic BNIP3^KD^ B16-F10 cells did secrete increased amounts of the pro-macrophage chemokine, CCL3, and the pro-inflammatory cytokine, IL1β (Supplementary Figure 2) [33].

**Figure 2:**
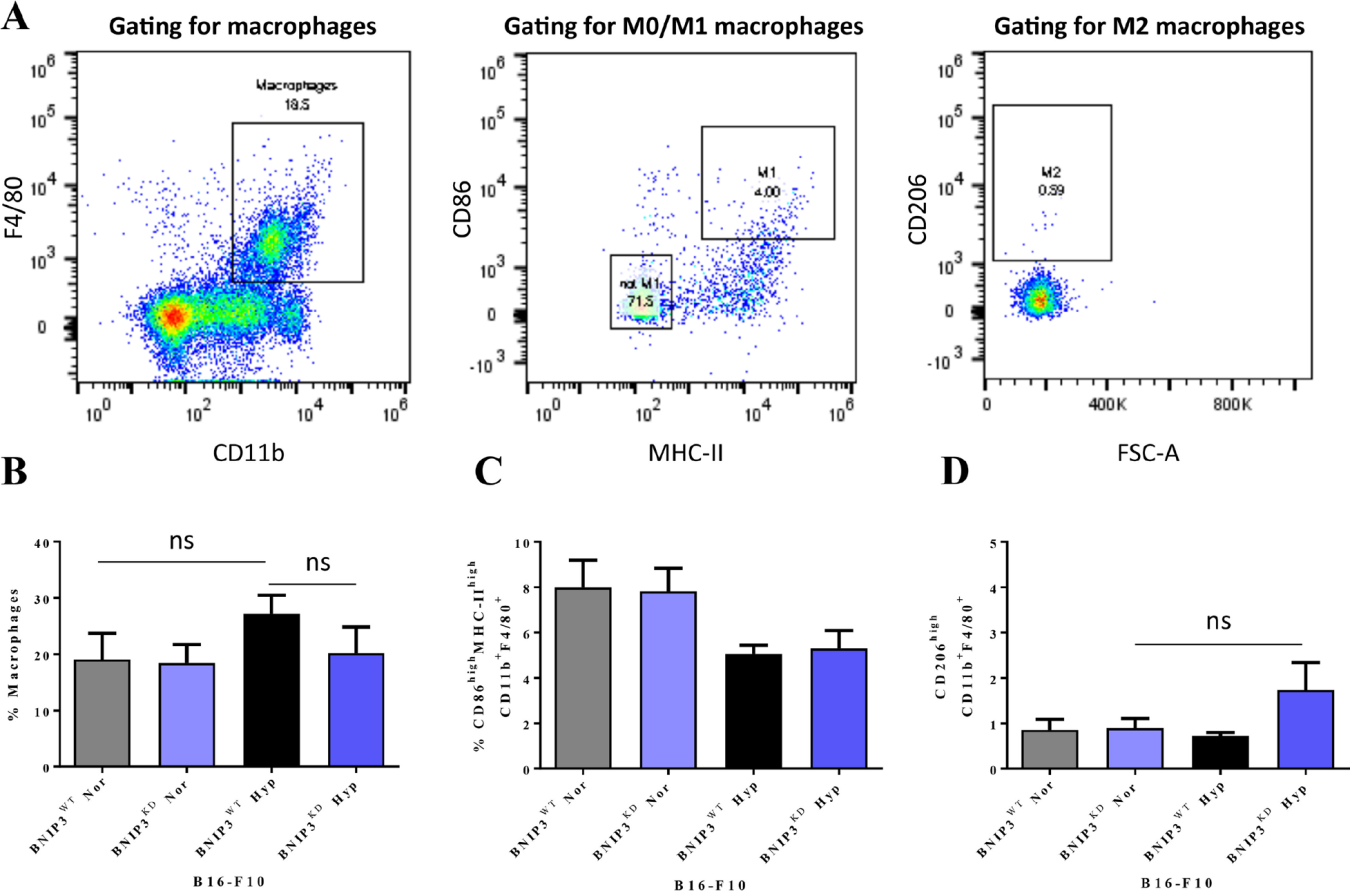
Ablation of BNIP3 and/or hypoxic pre-conditioning in melanoma cells has trivial consequences for the recruitment and polarization of macrophages *in vivo* (**A**) Gating strategy for the flow cytometry-based identification of the macrophage population and its polarization statuses. Quantification of (**B**) the recruitment of macrophages (F4/80^+^CD11^+^) in the peritoneum of mice, following injection of BNIP3^WT^ and BNIP3^KD^ B16-F10 cells kept under normoxia or hypoxia conditions, and their polarization towards (**C**) M1 (CD86^high^MHC-II^high^) or (**D**) M2 (CD206^high^) phenotype expressed as mean ± SEM (*n* = 6 mice/condition), all analysed with one-way ANOVA (ns = not significant; *p*-values from left to right: *p* = 0.50, *p* = 0.62 and *p* = 0.40)).

Next, to understand whether these results might translate into differential recruitment (and polarization) of macrophages *in vivo*, we utilized the murine peritoneal injection model, a well-established model to study myeloid chemotactic recruitment towards dying cells [34]. Herein, we injected the BNIP3^WT^ or BNIP3^KD^ B16-F10 cells, pre-treated or not with hypoxia, in the mice peritoneum. This was followed by flow cytometry-based analyses of peritoneal lavage-derived CD11b^+^F4/80^+^ macrophages (Figure 2A). Here, only hypoxic BNIP3^WT^ B16-F10 cells caused some, albeit insignificant (Figure 2B; *p* = 0.50), increase in macrophage recruitment. However, this increase was abolished in mice injected with hypoxic melanoma cells harbouring BNIP3^KD^ (Figure 2B; *p* = 0.62 vs. hypoxic BNIP3^WT^ cells).

We also analysed the phenotypic polarization status of peritoneal macrophages possibly interacting with the injected melanoma cells, owing to some interesting patterns observed above with pro-inflammatory cytokines. Macrophages tend to exist in various states of polarization depending on the context however there are three major polarization statuses i.e. M0 (naive, unpolarised macrophages; marked by CD86^low^MHC-II^low^CD206^low^CD11b^+^F4/80^+^), M1 (pro-inflammatory macrophages; marked by CD86^high^MHC-II^high^CD11b^+^F4/80^+^) and M2 (anti-inflammatory macrophages; marked by CD206^high^CD11b^+^F4/80^+^) (Figure 2A) [35]. In our settings, injection of different melanoma cell lines did not cause any statistically significant increase in the M1 polarization markers on the peritoneal lavage-derived macrophages (Figure 2C). Of note, we did observe a trend towards decrease in M1 macrophage polarization amongst mice injected with hypoxia pre-conditioned B16-F10 cells (irrespective of BNIP3 status), yet these trends were not statistically significant. On the level of M2 polarization markers however, the combination of BNIP3^KD^ in B16-F10 cells and hypoxic pre-conditioning, did potentiate (to a small, albeit insignificant extent; *p* = 0.40) the emergence of M2 macrophages (Figure 2D). However, these macrophage recruitment and polarization trends were not accompanied by any palpable increase in phagocytosis of (normoxic or hypoxic pre-conditioned) BNIP3^KD^ B16-F10 cells by the peritoneum-resident macrophages (Supplementary Figure 3).

### BNIP3 ablation impairs the secretion of ATP, a prototypical ICD-associated danger signal, from dying B16-F10 melanoma cells

Since the ablation of BNIP3 in B16-F10 cells, in combination with hypoxia, exerted only minor effects on the phagocytic activity and overall macrophage activation *in vivo*, we evaluated whether BNIP3’s immunomodulatory functionalities might be more evident in a cell death setup. Henceforth, we decided to study the effects of BNIP3 on chemotherapy-based ICD induction in B16-F10 melanoma cells.

**Figure 3:**
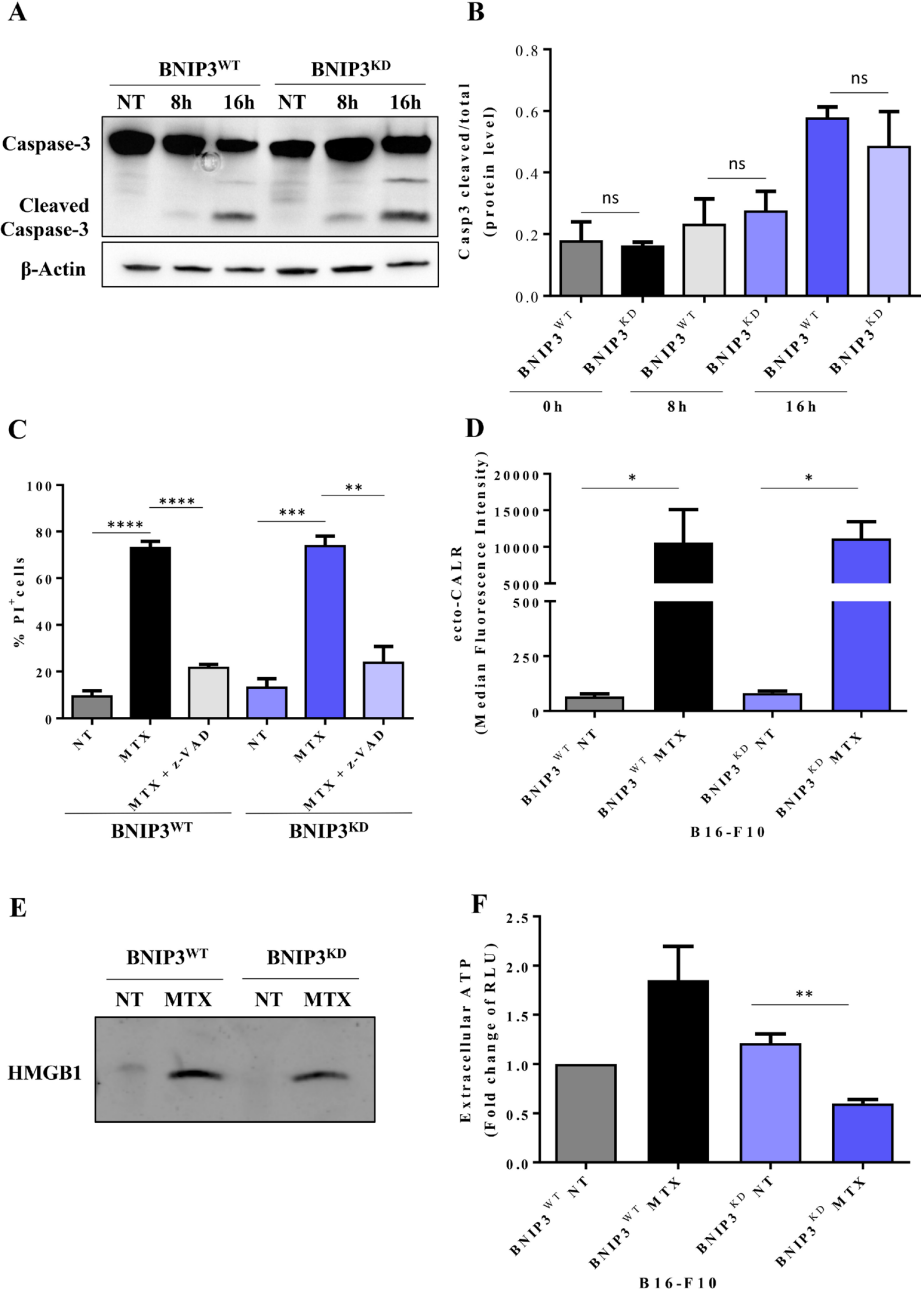
The secretion or surface exposure of danger signals in B16-F10 cells dying via apoptosis is partially affected by BNIP3 ablation (**A**) Representative immunoblot of caspase 3 cleavage in BNIP3^WT^ and BNIP3^KD^ B16-F10 cells after MTX-treatment at the indicated time points (NT: not treated). Actin was used as loading control. (**B**) Quantification of immunoblots probed with anti-Caspase 3 total and cleaved of BNIP3^WT^ and BNIP3^KD^ cells at different time-points (0 h, 8 h, 16 h). Data are expressed as mean ± SEM and analysed with Student’s *t*-test (ns = not significant, *n* = 3 independent experiments) (**C**) Cell death of B16-F10 (PI^+^) cells after 24 h treatment with MTX and pre-/co-incubation with z-Vad-fmk measured by flow cytometry. Data are expressed as mean ± SEM and analysed with Student’s *t*-test (^**^p = 0.0036, ^***^p = 0.0005, ^****^p < 0.0001, *n* = 3 biological replicates). (**D**) BNIP3^WT^ and BNIP3^KD^ B16-F10 cells were evaluated for surface exposure of CALR 16 h after MTX treatment in non-permeabilized cells via flow cytometry. Data are expressed as mean ± SEM and analysed with Mann-Whitney’s *t*-test (^*^p = 0.0286, *n* = 4 independent experiments). (**E**) Representative immunoblot for HMBG1 released in concentrated conditioned medium 48 h after treatment of BNIP3^WT^ and BNIP3^KD^ B16-F10 cells with MTX. (**F**) BNIP3^WT^ and BNIP3^KD^ B16-F10 were treated with MTX and conditioned media (24 h post treatment) were analysed for the presence of ATP. Data are expressed as fold change of RLU (Relative Light Units) with respect to untreated BNIP3^WT^ (mean ± SEM) and analysed with Student’s *t*-test (^**^p = 0.0035, *n* = 3 independent experiments).

To induce ICD, we treated B16-F10 melanoma cells with a *bona fide* ICD inducer i.e. the chemotherapeutic, mitoxantrone (MTX) [36]. Both BNIP3^WT^ and BNIP3^KD^ melanoma cells treated with MTX experienced a time-dependent increase in the cleavage of caspase-3 and cell death (Figure 3A–3C). However the overall caspase-3 cleavage wasn’t significantly altered between the two conditions (Figure 3B). In line with this, analyses at 24 h showed no significant differences in overall cell death between BNIP3^KD^ and BNIP3^WT^ cells (Figure 3C). Moreover, the pan-caspases inhibitor, zVAD-fmk, that typically inhibits caspases-dependent apoptosis [37] significantly reduced MTX-induced killing of both BNIP3^WT^ and BNIP3^KD^ melanoma cells (Figure 3C). This underscores the apoptotic nature of MTX-induced cell death in this setting.

**Figure 4:**
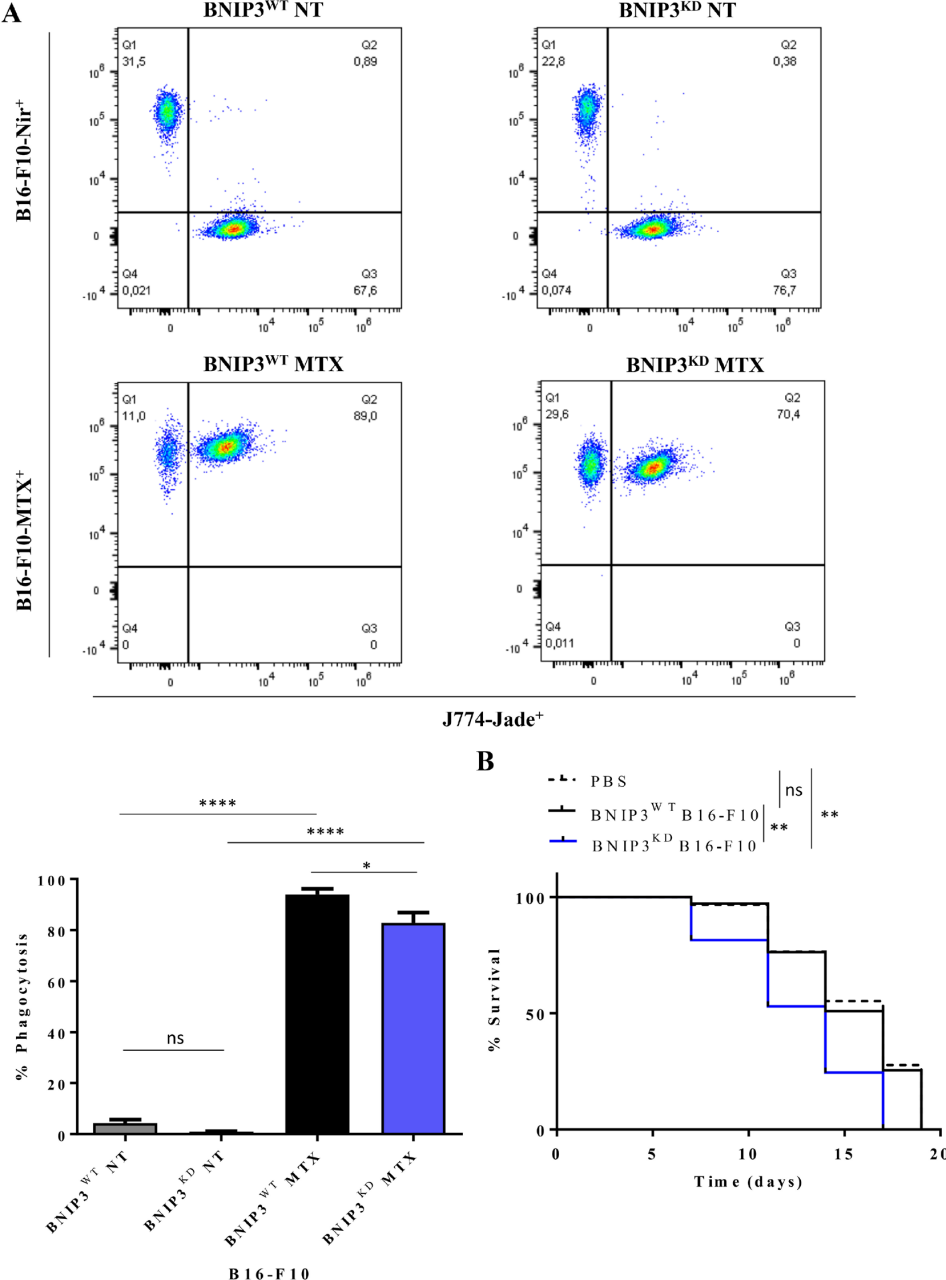
BNIP3 affects the phagocytic clearance of cells dying via ICD and decreases the intrinsic resistance of B16-F10 to ICD-mediated anticancer vaccination (**A**) Representative flow cytometry gates of J774-mediated phagocytosis of B16-F10 treated with MTX after 2 h co-incubation (upper panel) and relative quantification (lower panel) of B16-F10-Nir^+^ or B16-F10-MTX^+^ and J774-Jade^+^ cells. Data expressed as mean ± SEM and analysed with Student’s *t*-test (^*^p = 0.034, ^****^p < 0.0001, ns = not significant, *n* = 2 independent experiments). (**B**) Survival curve of C57BL/6 mice vaccinated with BNIP3^WT^ or BNIP3^KD^ B16-F10 cells treated with MTX or PBS control. Data are analysed with log-rank/Mantel-Cox test (^**^p < 0.05, ns = not significant, *n* = 7 mice/condition).

Next, we assessed the three major danger signals associated with ICD, that also serve as surrogate biomarkers of this process i.e. ecto-CALR, secreted ATP and released HMGB1 [36]. Indeed, B16-F10 melanoma cells treated with MTX emitted all 3 danger signals (Figure 3D–3F), in line with previous reports [38]. Interestingly, BNIP3 ablation in melanoma cells profoundly reduced (^**^p = 0.0035) the secretion of ATP (Figure 3F), without significantly affecting the emission of the other two danger signals (Figure 3D, 3E). Finally, in line with the increased ecto-CALR (Figure 3D) following MTX treatment, BNIP3^WT^ B16-F10 cells treated with MTX underwent rapid phagocytosis by the J774 cells (Figure 4A). Intriguingly, in this setting, MTX-treated BNIP3^KD^ B16-F10 cells exhibited a slight but significant reduction (^*^p = 0.034) in the phagocytic clearance, thereby further suggesting a pro-ICD role for BNIP3. Thus, based on the analyses of surrogate ICD markers indicating a BNIP3-dependent ATP secretion and phagocytic clearance of dying/dead cells, BNIP3 may have a pro-ICD function in B16-F10 cells.

### The intrinsic resistance of B16-F10 to ICD-mediated anticancer vaccination is potentiated by BNIP3 ablation

The trends observed above with the prototypical ICD-associated danger signals, especially the BNIP3-secreted ATP link, motivated us to unravel the putative effects of BNIP3 on ICD-associated anticancer vaccination effect *in vivo*.

MTX-treated BNIP3^WT^ or BNIP3^KD^ B16-F10 cells were injected subcutaneously into the right flank of syngeneic immune-competent C57BL/6 mice (with PBS injected mice serving as controls). Post-vaccination, these rodents (including the PBS injected ones) were re-challenged with live B16 cells in the opposite flank. Thereafter, protection against tumour growth at the re-challenge site was interpreted as a sign of anticancer vaccination effect and expressed as tumour-free survival. To our surprise, none of the mice vaccinated with MTX treated B16-F10 cells exhibited tumour-rejecting responses (Figure 4B) - a sign of ICD resistance [26]. Interestingly, mice injected with MTX-treated BNIP3^KD^ B16-F10 succumbed to tumour re-challenge significantly faster (^**^p < 0.05) than those injected with PBS or MTX-treated BNIP3^WT^ B16-F10 cells (Figure 4B).

In conclusion, B16-F10 melanoma cells have an intrinsic ICD-resistant phenotype that is further potentiated by ablation of BNIP3 thereby advocating a partial pro-ICD role for BNIP3.

## DISCUSSION

In this study, we aimed to evaluate the impact of BNIP3 ablation in melanoma cells, on the interface with immune cells on various levels particularly, phagocytosis, chemotaxis and functional polarization (all three for macrophages), as well as susceptibility to ICD. In general, we found that BNIP3 modulates melanoma cell-immune cell intersection in a highly contextual fashion and in a manner contingent upon the plasticity and immunoevasive character of the melanoma cells. For instance under normoxic conditions, despite having very low ecto-CD47, the BNIP3^KD^ B16-F10 melanoma cells were not readily phagocytosed by macrophages. Hypoxic treatment was required to accentuate these cells’ phagocytic clearance. Moreover, although hypoxia elicited an increase in phagocytosis, this did not seem to be contingent upon the differential ecto-CD47 levels. We also ruled out the role of ecto-CALR (on account of its absence), a notorious ‘eat me’ signal known to counter-balance ecto-CD47’s function [21]. These observations in melanoma cells stand in contrast to previous studies showing hypoxic or stressed cancer cells escaping from macrophage-based phagocytosis by increasing ecto-CD47 [15, 20, 21, 39]. This further highlights the complexity of the melanoma-elicited immunosuppressive paradigm. Taken together, our observations suggest that hypoxia might have a variable role in regulating phagocytic clearance depending upon the cancer-type under consideration. Nevertheless, the apparent dispensability of CD47 and the absence of ecto-CALR, despite on-going phagocytosis, may be a sign of B16-F10 melanoma cells utilizing either alternative ‘don’t eat me’ signals or highly potent ‘eat me’ signals capable of circumventing ecto-CD47. Although ecto-CD47 is a very ubiquitous anti-phagocytic signal [15], yet there have been instances of other ‘don’t eat me’ signals playing a more dominant anti-phagocytic role, like CD200 or the MHC class I-LILRB1 signalling axes [16, 40, 41]. On the contrary, beyond ecto-CALR, not many ‘eat me’ signals have been systematically reported to counter-balance ecto-CD47 [16]. These gaps in knowledge need to be addressed in near future through an explorative screening study. Finally, with respect to hypoxia’s pro-phagocytic effects, whereas hypoxic environment facilitated phagocytic clearance of B16-F10 cells *in vitro*, yet hypoxic pre-conditioning of the same cells failed to accentuate phagocytic clearance *in vivo*. At the outset, we believe this might be a result of differences in hypoxic persistence. While in the *in vitro* setting, hypoxia persisted throughout the duration of phagocytosis, in the *in vivo* setting it was systematically exerted only before the injection of the cells in the mouse peritoneum. It is conceivable that post-injection, the same level of hypoxia may not have been maintained *in vivo*, hence explaining the *in vitro* vs. *in vivo* differences in phagocytosis [42, 43].

We also observed a failure in recruiting macrophages by the melanoma cells *in vivo*, which could be due to the following reasons: (1) the overall levels of chemokines secreted by melanoma cells may have failed to reach the thresholds required to recruit macrophages from the circulation into the peritoneum; or, (2) the mouse peritoneum is a relatively large area hence, through lavage analyses, it may not be possible to appreciate the directional re-orientation of peritoneum-resident macrophages towards the specific site of injection. However, BNIP3 proficient or deficient melanoma cells injected into the transgenic zebrafish larvae expressing mCherry^+^macrophages (*fms:nfsB.mCherry*; to analyse localized chemotaxis close to injection site via intra-vital microscopy [27, 44]), also failed to recruit the macrophages (data not shown). This suggests that BNIP3 presence does not affect the ability of living melanoma cells to recruit these innate immune cells, at least in these *in vivo* settings. Nevertheless, these results outline the extremely variable nature of the immunological interface in an *in vivo* context.

Remarkably, the above immunoevasive behaviour of B16-F10 cells was also visible in a therapeutic cell death setting. More specifically, despite the ability of these cells to proficiently emit prominent danger signals like ecto-CALR, secreted ATP and passively released HMGB1 after treatment with the *bona fide* ICD inducer MTX, B16-F10 cells were unable to elicit anticancer vaccination effect *in vivo*. In past, others and we have demonstrated that, naturally occurring or artificially-induced, defects in ecto-CALR exposure, ATP secretion or HMGB1 release can cause cancer cells to resist the pro-immunogenic effects of ICD [25, 26, 28, 45–48]. However, B16-F10 cells proficiently emitted these danger signals thereby indicating existence of other immunoresistance pathways that need to be identified in the near future [49, 50]. It also needs to be identified whether only B16-F10 melanoma cells are resistant to ICD *in vivo*, or also other known murine melanoma cellular models phenocopy this [38, 45, 51–52]. To address this, ICD-relevant analyses should be extended to more melanoma models, in not only transplantable but also spontaneous or autochthonous settings [38]. Such analyses could be crucial to understand whether melanoma has an intrinsic inability to undergo ICD *in vivo* or *in situ*, an interesting notion that may explain the limited success of ICD-inducing chemotherapeutics like anthracyclines against melanoma in the clinic [45].

Nevertheless, in the ICD set-up, it seemed that BNIP3 plays a partial pro-immunogenic role. More specifically, BNIP3^KD^ in B16-F10 cells caused significantly lower secretion of ATP and slightly (but significantly) impaired phagocytic clearance of dying/dead B16-F10 cells. Moreover, mice vaccinated with dead/dying BNIP3^KD^ B16-F10 cells exhibited significantly reduced survival when compared to those vaccinated with dead/dying BNIP3^WT^ B16-F10 cells. It is highly conceivable that the strong reduction in ATP secretion may account for this reduction in survival [53]. Of note, it may not be surprising that BNIP3 ablation affects the secretion of ATP. BNIP3 is a well-known pro-autophagic/pro-mitophagic protein [10] and autophagy has been widely reported to play a pivotal role in secretion of ATP following MTX [54–56]. Based on our results, it would be interesting to explore in the future whether mitophagy is the dominant autophagic (sub-)pathway for ATP secretion following chemotherapy. Secretion of ATP by BNIP3^KD^ B16-F10 cells under basal (untreated) conditions, was not reduced as compared to the untreated BNIP^WT^ cells but was even slightly increased, albeit insignificantly and to an extent that is likely not biologically relevant. However, it should be mentioned that extracellular ATP secretion pathways are highly context-dependent, and thus differences may exist in ATP secretion under basal conditions as compared to specific stressed conditions. Last but not least, the partial pro-phagocytic effects of B16-F10 cells-associated BNIP3 in this cell death set-up were intriguing in the light of no apparent changes in ecto-CALR. It would be important to understand in the near future, whether this indicates the presence of an alternate BNIP3-regulated ‘eat me’ signal.

In conclusion, we found that melanoma cell-associated BNIP3 played a highly contextual immunomodulatory role at the melanoma-immune cell interface. On one hand, in living melanoma cells BNIP3 seemed to affect immunomodulatory responses elicited by hypoxia signalling. Hence, we believe that future studies should strive to reveal the full relevance of BNIP3 using appropriate melanoma mice models recapitulating the hypoxic tumour microenvironment. On the other hand, in response to ICD, BNIP3 accentuated ATP secretion, phagocytic clearance and the vaccination potential of the dying melanoma cells. However, we failed to fully appreciate the pro-ICD impact of BNIP3 due to the intrinsic resistance of B16-F10 cells to ICD-associated anticancer vaccination effect *in vivo*. It would be interesting in the future to re-examine the role of BNIP3 in cancer models susceptible to immunosurveillance and ICD.

## MATERIALS AND METHODS

### Cell culture

B16-F10 were cultured at 37° C, either under 5% CO2 and 20% O2 (Normoxia) or 5% CO2 and 1.5% O2 (Hypoxia), in RPMI medium (Sigma, R8758) supplemented with 10% FBS, 2 mM glutamine, 100 units/ml penicillin, 0.1 mg/ml streptomycin. Generation of shRNA stable clones was performed as previously described [13]. Briefly, the shRNAs targeted against the murine mRNA coding for BNIP3 were cloned in the lentiviral pLKO.1-puro vector (Sigma-Aldrich, St. Louis, MO, USA). An empty pLKO.1-puro control vector was used as a control (CTL) (BCCM/LMBP Plasmid collection). To generate lentiviral particles, HEK 293T cells were transfected by the calcium phosphate method with 4 mg of pLKO.1-puro carrying the shRNAs or with empty pLKO.1-puro. The viral vectors were then added to the exponentially growing B16-F10 cell cultures in the presence of 8 mg/ml of polybrene. The cells were expanded and selected via puromycin (9 mg/ml) for 3 days.

Murine macrophages J774 were kindly provided by S. van den Brule (UCL) and cultured in DMEM medium and supplemented with 10% FBS, 2 mM glutamine, 100 units/ml penicillin and 0.1mg/ml streptomycin.

### Cell death induction and quantification

Mitoxantrone (MTX) was purchased from Sigma-Aldrich (M6545) and used at a concentration of 8 µM to induce immunogenic cell death. Apoptosis induction was counteracted by pre-treating for 4 h with the pan-caspases inhibitor carbobenzoxy-valyl-alanyl-aspartyl-[O-methyl]-fluoromethylketone (zVAD-fmk, 50 μM) purchased from Bachem (N1560).

To estimate the amount of cell death, supernatant and cells were collected with TrypLE Express and resuspended in PBS containing propidium iodide (PI) (Sigma, P4170). Samples were subsequently acquired via Attune Flow Cytometer (Life Technologies) and data analysis was performed via FlowJo_V10^™^ software (Tree Star, Ashland, OR, USA).

### Flow cytometry-based detection of cell surface CD47 and CALR

B16-F10 cells were washed with PBS, detached with TrypLE Express (ThermoFisher, 12604–21), centrifuged and washed once with ice-cold Flow Cytometer (FC) buffer (2% BSA, 1% FBS in PBS). For the detection of CD47, cells were incubated with FITC-conjugated anti-CD47 (ThermoFisher, 11-0471-82) in FC buffer at 4° C for 45 min. For the detection of ecto-CALR instead, cells were incubated with anti-CALR (Abcam, Ab92516) at 4° C, for 40 min, in FC buffer followed by Alexa Fluor647 Anti-Mouse IgG (ThermoFisher, A-27040) at 4° C, for 40 min. Then the cells were resuspended in FC buffer including 1 µmol/L Sytox Green (Life Technologies, S7020). Due to the high autofluorescence of MTX, the background fluorescent measured in the unstained sample was subtracted from the median fluorescent intensity of the relative stained samples. Cells were acquired with Attune Flow Cytometer and data analysis was performed via FlowJo_V10^™^ software.

### *In vitro* phagocytosis assay

B16-F10 and J774 were detached with TrypLE Express and labelled respectively with CellVue^®^Nir780 (Affymetrix eBioscience, 88–0875) and CellVue^®^Jade (Affymetrix eBioscience, 88–0876). J774 were kept in serum-free medium for 4 h and subsequently co-incubated with B16-F10 at a 1:5 ratio (Macrophages : Cancer Cells) for 24 h, under both normoxic and hypoxic conditions.

Alternatively B16-F10 were treated with MTX and co-incubated for 2 h with J774 at a 5:1 ratio (Macrophages : Cancer Cells). The autofluorescence of MTX was used as label of cancer cells. Cells were harvested using TrypLE Express and acquired with Attune Flow Cytometer. Data analysis was performed via FlowJo_V10^™^ software.

### Immunoblotting

Cells were lysed in a buffer containing 100 mM Hepes 7.4, 10% sucrose, 1% Triton ×100, 2.5 mM EDTA, 5 mM DTT, 1 mM PMSF, 2 µg/ml pepstatin, and 2 µg/ml leupeptin. For BNIP3 extraction instead, cells were lysed with a modified Laemmli buffer [13]. Proteins were separated by SDS-PAGE under reducing conditions, transferred to a nitrocellulose membrane and analysed by immunoblotting. Primary antibodies detecting BNIP3 (Biokè, 3769S), total (Biokè, 9662S) and cleaved (Biokè, 9661S) caspase 3, HMGB1 (Genetex, GTX12029) and β-actin (Sigma) were further detected with the appropriate horse radish peroxidase-conjugated secondary antibody (Thermo Scientific), whose reaction was in-turn incited with Pierce ECL Western Blotting Substrate (ThermoFisher, 32106). Immunoblotting for concentrated conditioned media-associated HMBG1 was carried out as reported before [27]. Quantification of the images was performed via ImageJ.

### Antibody array

B16-F10 proficient and deficient for BNIP3 were kept under normoxia or hypoxia and after 24h the supernatant was collected, centrifuged and processed according to manufacturer’s instructions (R&D systems, ARY015). The membranes were scanned using the Bio-Rad Chemidoc Imager (Bio-Rad Laboratories N.V.3, Winninglaan, Temse, Belgium) while ImageJ was used for the quantification.

### ATP assay

The cells were treated as indicated. Extracellular ATP was measured in the conditioned medium (2% FBS) via an ATP Bioluminescent assay kit (Sigma) based on luciferin-luciferase conversion, following manufacturer’s instructions. Bioluminescence was assessed by optical top reading via FlexStation 3 microplate reader (Molecular Devices Inc., Sunnyvale, CA, USA).

### Prophylactic mouse vaccination

Mouse experiments were performed at KU Leuven (Leuven, Belgium) in the designated animal facilities and in accordance with the institutional and national guidelines and regulations. Animals were purchased from the internal stock of the animal facility at KU Leuven, Belgium. The prophylactic vaccination was performed in immunocompetent syngeneic (C57/Bl6) female mice (6–9 weeks old), via subcutaneous injection of 200 µL PBS with dying B16-F10 cells (3 × 10^6^ cells in 200 µL PBS) or 100 µL of PBS only into the right flank, two-times in a time frame of 2-weeks. After 7 days from the second injection, mice were re-challenged with untreated BNIP3^WT^ B16-F10 cells into the left flank (5 × 10^5^ cells in 100 µL PBS) and tumour growth was monitored for 20 days.

### *In vivo* phagocytosis assay

C57BL/6 female mice (6–9 weeks old) were injected intra-peritoneum with PBS containing 3 × 10^6^ B16-F10 cells (BNIP3^WT^ or BNIP3^KD^) pHrodo-labelled (Life Technologies, P36600). The peritoneal cells were collected 24 h post-injection via peritoneal lavage with Ca^2+^- and Mg^2+^-free PBS. The cells were transferred to an ultra-low attachment V-bottom plate and stained with the fixable Live/Dead Yellow stain (Invitrogen). After blocking the F_c_ receptor (CD16/32, eBioscience, 16-0161-82), cells were stained for F4/80-eFluor780 (eBioscience, 47-4801-80), CD11b-eFluor450 (eBioscience, 48-0112-82), MHCII-FITC (eBioscience, 11-5321-81), CD86-APC (eBioscience, 17-0862-81), CD206-PeCy7 (eBioscience, 25-2061-80) in FC buffer. Samples were acquired on a Gallios^™^ flow cytometer and the data analysis was performed via FlowJo_V10^™^ software.

### Statistical analysis

The statistical analyses were performed via GraphPad Prism 6 (Graphpad Software, San Diego, CA, USA) and are indicate in the respective figure legends. Grubbs’ test was used to exclude outliers, where applicable.

## SUPPLEMENTARY MATERIALS FIGURES



## Abbreviations

ATP: adenosine triphosphate
BNIP3: Bcl-2 [B-cell Leukaemia/Lymphoma 2]/Adenovirus E1B1] Nineteen kD Interacting Protein 3
CALR: calreticulin
CD: cluster of differentiation
GM-CSF: granulocyte-macrophage colony stimulating factor
HMGB1: high-mobility group box-1
ICD: immunogenic cell death
IL: interleukin
MTX: mitoxantrone
