# Correction: BNIP3 modulates the interface between B16-F10 melanoma cells and immune cells

**DOI:** 10.18632/oncotarget.26474

**Published:** 2018-12-11

**Authors:** Erminia Romano, Nicole Rufo, Hannelie Korf, Chantal Mathieu, Abhishek D. Garg, Patrizia Agostinis

**Affiliations:** ^1^ Laboratory for Cell Death Research and Therapy (CDRT), Department of Cellular and Molecular Medicine, KU Leuven, Leuven, Belgium; ^2^ Laboratory of Hepatology, Department of Chronic Diseases, Metabolism and Ageing (CHROMETA), KU Leuven, Leuven, Belgium; ^3^ Laboratory of Clinical and Experimental Endocrinology (CEE), Department of Chronic Diseases, Metabolism and Ageing (CHROMETA), KU Leuven, Leuven, Belgium

**This article has been corrected:** During the assembly of the Figure lB, the flow cytometry histogram concerning ecto-CD47 expression of B16-F10 cell lines exposed to normoxic (Nor) conditions was presented incorrectly. Using the source data, a correct Figure 1B was generated and is shown below. The authors declare that these corrections do not change the results or conclusions of this paper.

**Figure 1:**
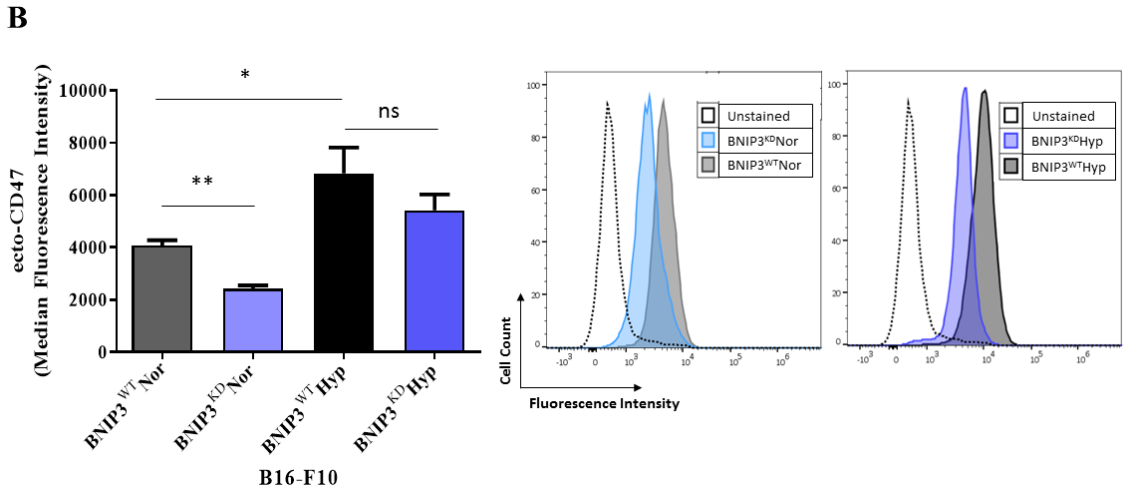
BNIP3 and hypoxia modulate the phagocytosis of B16-F10 melanoma cells by macrophages (B) Flow Cytometry-based quantification (left panel) and representative histograms (right panel) of the level of surface CD47 (ecto-CD47) in B16-F10 cells (BNIP3^WT^ and BNIP3^KD^) after 24 h of culture in normoxia (Nor) or hypoxia (Hyp). Data expressed as mean ± SEM and analysed with Mann-Whitney's *t*-test (**p = 0.0043, *p = 0.0411, ns = not significant [*p* = 0.3052]) as indicated by the bar, *n* = 3 independent experiments).

Original article: Oncotarget. 2018; 9:17631-17644. https://doi.org/10.18632/oncotarget.24815

