# Supplementary material for: BNIP3 modulates the interface between B16-F10 melanoma cells and immune cells
Source: Oncotarget. 2018 Apr 3;9(25):17631–44. doi: 10.18632/oncotarget.24815 (PMC5915144; doi:10.18632/oncotarget.24815)
Supplement: Supplementary file 1 [file oncotarget-09-17631-s001.pdf]

BNIP3 modulates the interface between B16-F10 melanoma cells and immune cells

SUPPLEMENTARY MATERIALS

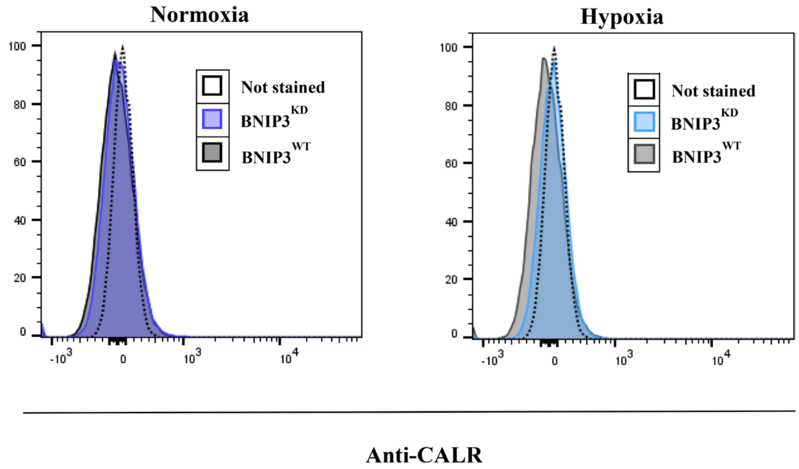

Supplementary Figure 1: Representative histograms of the levels of ecto-CALR on BNIP3^{WT} and BNIP3^{KD} B16-F10 cells kept 24 h under normoxia or hypoxia

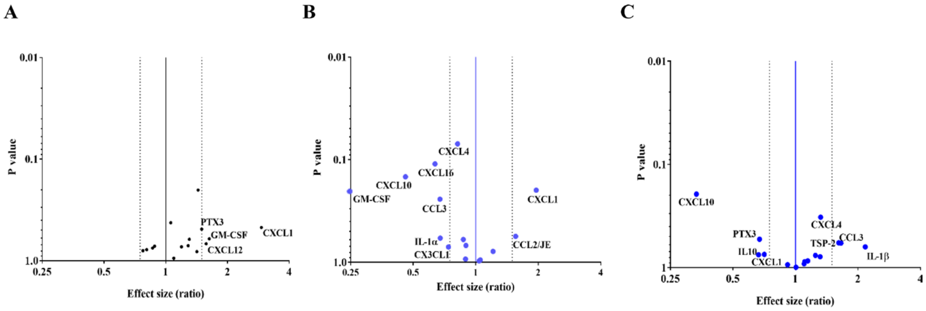

Supplementary Figure 2: BNIP3 and hypoxia differentially regulate secretion of pro-macrophage chemokines and pro-inflammatory cytokines by B16-F10 melanoma cells. Volcano plots depicting the changes (indicated by the increased or decreased effect size [ratio]) in the major murine chemokines detected in the secretome of BNIP3^{WT} and BNIP3KD B16-F10 cells measured with an antibody array. The conditions compared are: (**A**) BNIP3^{WT} under hypoxia versus normoxia; (**B**) BNIP3^{KD} versus BNIP3^{WT} all under normoxia; (**C**) BNIP3KD versus BNIP3WT all under hypoxia. The 1.5 effect size (ratio) increase or 0.75 effect size (ratio) decrease in secretion/release is indicated by the dotted lines (n = 3, Student's t-test).

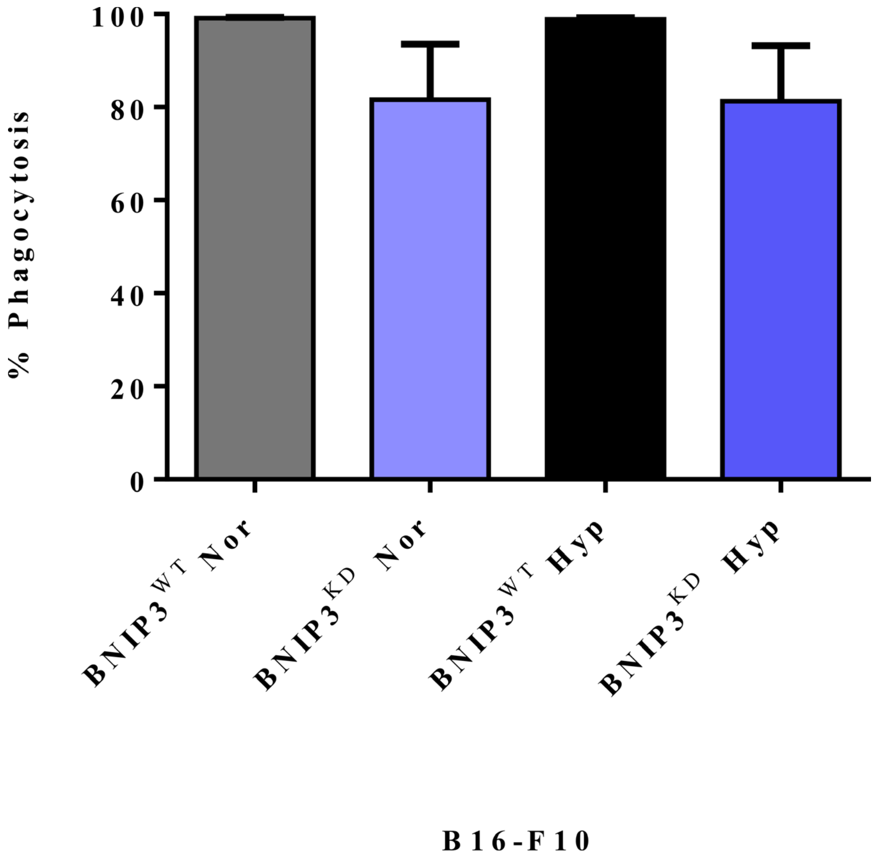

Supplementary Figure 3: Phagocytosis of BNIP3^{KD} B16-F10 cells is not increased *in vivo*. Quantification via flow cytometry of BNIP3^{KD} or BNIP3^{WT} B16-F10 cells phagocytosed by intraperitoneal macrophages in C57BL/6 mice after 24 h pre-conditioning in normoxia or hypoxia. The engulfing ability was expressed as percentage of F4-80+/CD11b+/pHRodo+ cells (mean ± SEM, mice = 6/ group, Student's *t*-test).
